# Intraperitoneal Administration of a Tumor-Associated Antigen SART3, CD40L, and GM-CSF Gene-Loaded Polyplex Micelle Elicits a Vaccine Effect in Mouse Tumor Models

**DOI:** 10.1371/journal.pone.0101854

**Published:** 2014-07-11

**Authors:** Kouichi Furugaki, Lin Cui, Yumi Kunisawa, Kensuke Osada, Kentaro Shinkai, Masao Tanaka, Kazunori Kataoka, Kenji Nakano

**Affiliations:** 1 Innovation Center for Medical Redox Navigation, Kyushu University, Fukuoka, Japan; 2 Department of Surgery and Oncology, Graduate School of Medical Sciences, Kyushu University, Fukuoka, Japan; 3 Department of Materials Engineering, Graduate School of Engineering, The University of Tokyo, Tokyo, Japan; 4 Division of Clinical Biotechnology, Center for Disease Biology and Integrative Medicine, Graduate School of Medicine, The University of Tokyo, Tokyo, Japan; Mie University Graduate School of Medicine, Japan

## Abstract

Polyplex micelles have demonstrated biocompatibility and achieve efficient gene transfection *in vivo*. Here, we investigated a polyplex micelle encapsulating genes encoding the tumor-associated antigen squamous cell carcinoma antigen recognized by T cells-3 (SART3), adjuvant CD40L, and granulocyte macrophage colony-stimulating factor (GM-CSF) as a DNA vaccine platform in mouse tumor models with different types of major histocompatibility antigen complex (MHC). Intraperitoneally administrated polyplex micelles were predominantly found in the lymph nodes, spleen, and liver. Compared with mock controls, the triple gene vaccine significantly prolonged the survival of mice harboring peritoneal dissemination of CT26 colorectal cancer cells, of which long-term surviving mice showed complete rejection when re-challenged with CT26 tumors. Moreover, the DNA vaccine inhibited the growth and metastasis of subcutaneous CT26 and Lewis lung tumors in BALB/c and C57BL/6 mice, respectively, which represent different MHC haplotypes. The DNA vaccine highly stimulated both cytotoxic T lymphocyte and natural killer cell activities, and increased the infiltration of CD11c^+^ DCs and CD4^+^/CD8a^+^ T cells into tumors. Depletion of CD4^+^ or CD8a^+^ T cells by neutralizing antibodies deteriorated the anti-tumor efficacy of the DNA vaccine. In conclusion, a SART3/CD40L+GM-CSF gene-loaded polyplex micelle can be applied as a novel vaccine platform to elicit tumor rejection immunity regardless of the recipient MHC haplotype.

## Introduction

Cancer vaccines have attracted attention as a promising modality to treat patients with malignancies, because they elicit specific rejection immunity against tumor-associated antigens (TAAs) with minimal invasiveness to normal tissues in contrast to chemotherapy, irradiation, and surgery. During vaccination, fragmented TAA peptides bound to the major histocompatibility complex (MHC) expressed by antigen-presenting cells (APCs), such as dendritic cells (DCs), [1] stimulate naive T lymphocytes to mature into helper and cytotoxic T lymphocytes (CTLs) in concert with co-stimulatory signals via the B7/CD28 interaction [2]. Granulocyte macrophage colony-stimulating factor (GM-CSF) mobilizes DCs and upregulates the expression of MHC and B7 on DCs [3]. The CD40L interaction with CD40 on DCs is known to further mature DCs, resulting in an enhanced vaccination effect [4], [5].

Peptide-, cell- and gene-based vaccines have been applied for treatment of cancer and infection diseases in animal models and human clinical trials. Peptide vaccines have the advantages of low production costs, high safety, and good compliance for clinical application. However, it is difficult to identify which TAA-epitope peptides elicit strong vaccination effects against tumors with low immunogenicity [6], [7]. It is also necessary to match the epitope-peptide and MHC haplotype, resulting in limited eligibility of patients receiving the vaccination [6], [7]. For cell-based vaccines, TAA genes are transduced into DCs or autologous tumor cells *ex vivo* using viral vectors. Accordingly, production of cell-based vaccines is time consuming, less versatile for target modification, and highly costly because of biomaterial handling [8]. However, cell-based vaccines allow co-expression of TAA and adjuvant genes to induce more efficient rejection of weakly immunogenic TAAs. For example, GM-CSF- and CD40L-expressing DC vaccines have been evaluated in clinical trials [9]. Furthermore, a recent study has shown that tumor cell vaccines with CD40L and GM-CSF gene transduction have a higher therapeutic efficacy than that of tumor cell vaccines with transduction of each single gene [10]. However, whether direct transduction of these adjuvant genes affects the immunological response and contributes to TAA-specific tumor rejection *in vivo* is unknown.

Gene-based vaccines to induce anti-tumor immunity using non-viral vectors may resolve these issues and the safety concern of viral vectors. For *in vivo* gene transfection without severe tissue injury, polyplex micelles are an intriguing system [11]–[13], which are constructed by the self-assembly of poly(ethyleneglycol)(PEG)-polycation block catiomers and plasmid DNA (pDNA). Because of the characteristic core-shell compartmentalized architecture, in which pDNA is packaged within the core and surrounded by PEG as the shell, the functional genes are protected from interactions with biological components, resulting in substantial stability within the physiological environment. Recently, we found that intraperitoneally administrated polyplex micelles are preferentially distributed at tumors sites and in immune organs of mice harboring peritoneally disseminated cancer cells [14], [15]. This study prompted us to examine the vaccine effect and adjuvant mechanism for anti-cancer immunity *in situ* by transfection of a TAA gene and adjuvant GM-CSF/CD40L genes. In the current study, we used the homo-catiomer-integrated polyplex micelle system formulated by a multibiofunctional catiomer, poly{N′-[N-(2-aminoethyl)-2-aminoethyl]aspartamide}, P[Asp(DET)] (H), and its PEG conjugated form, PEG-P[Asp(DET)] (B), with an optimized B/H composition of 70/30 for superior efficiency and safety [16]. The BH polyplex micelle exhibits high transfection efficiency by promotion of cellular uptake and enhancement of the endosome escape function derived from the P[Asp(DET)] segment [17]. Furthermore, this micelle shows reduced cumulative cytotoxicity because of the self-catalytic degradation profile of the P[Asp(DET)] segment in the physiological environment [18], [19], thus retaining suitable properties for gene-based vaccination.

Squamous cell carcinoma recognized by T cell-3 (SART3) is involved in RNA splicing in various cancers but not in normal tissues [20]. Synthetic SART3 peptides bind to various mouse and human MHC haplotypes and exhibit immunogenicity as cancer vaccines in mouse tumor models and clinical studies [21]–[23]. In this study, we examined the potential of a non-viral polyplex micelle-based DNA vaccine in mouse tumor models with different MHC haplotypes. Intraperitoneal (i.p.) administration of polyplex micelles exhibited a vaccine effect via CD4/CD8a^+^ T cell-mediated immunity by co-transfection of SART3, CD40L, and GM-CSF genes. Thus, a TAA/CD40L+GM-CSF gene-loaded polyplex micelle may be a promising vaccine platform for recipients with any MHC haplotype.

## Materials and Methods

### Plasmid DNA construction

Expression plasmids for GM-CSF, CD40L, or SART3 genes were constructed as follows. The open reading frames of mouse GM-CSF, CD40L, or SART3 genes (accession numbers BC116880.1, NM_011616.2, and NM_016926.1, respectively) were integrated at multi-cloning sites in a pVIVO1-mcs2 plasmid (InvivoGen, San Diego, CA). The plasmids were amplified in *Escherichia coli* DH5A competent cells and purified using an EndoFree Plasmid Giga Kit (Qiagen, Valencia, CA).

### Preparation and characterization of polyplex micelles

The homo-catiomer of P[Asp(DET)] [H, degree of polymerization (DP): 55] and block-catiomer of PEG-*b*-P[Asp(DET)] (B, *M_w_* of PEG: 12000; DP: 65) were kindly provided by NOF Corp. (Kawasaki, Japan). The BH polyplex micelle was prepared as described elsewhere [16]. Briefly, polymer solutions of B and H, which were dissolved in 10 mM HEPES buffer (pH 7.3), were mixed at a B/H ratio of 70/30 at their residual molar ratio of amino groups. Then, the mixed polymer solution was added to a solution of pDNA in 10 mM HEPES buffer (pH 7.3) for complexation at an N/P ratio (residual molar ratio of total amino groups in B and H to phosphate groups in pDNA) of 10 to obtain the BH polyplex micelle.

The ζ-potential of the BH polyplex micelle was measured by an ELSZ-2 (Otsuka Electronics, Osaka, Japan) at 25°C. The size and polydispersity index (PDI) of the polyplex micelle were evaluated by measurement of the dynamic light scattering (DLS) at 25°C using the ELSZ-2 equipped with a He-Ne ion laser (633 nm) with the incident beam at a detection angle of 160° as reported previously [14].

### Cell lines

Mouse colorectal carcinoma (CT26), lymphoma (YAC-1), Lewis lung carcinoma (3LL/LLC), and human pancreatic cancer SUIT2 cells were obtained from the American Type Culture Collection. The cells were maintained in RPMI 1640 medium (Nacalai Tesque, Kyoto, Japan) supplemented with 10% heat-inactivated fetal bovine serum (FBS, Wako Pure Chemical Industries, Osaka, Japan), 100 U/ml penicillin, and 100 µg/ml streptomycin at 37°C in a humidified incubator containing 5% CO_2_.

### Animals

BALB/c AnNCrlCrlj and C57BL/6J mice (female, 6 weeks old) were purchased from Charles River Laboratories (Yokohama, Japan). Animals were housed in a temperature-controlled room under a 12/12 hour light/dark cycle with free access to food and water. All animal procedures were approved and carried out in accordance with the Institutional Guidelines for Animal Experiments of the Animal Care and Use Committee at Kyushu University.

### Polyplex micelle distribution after i.p. administration

PEG-*b*-P[Asp(DET)] was labeled with Fluolid-orange fluorescence (kindly provided by Dr. Takaaki Kanemaru, Kyushu University) as reported previously [14]. Fluorescence-labeled PEG-*b*-P[Asp(DET)]/P[Asp(DET)] mixed micelles with pVIVO-1-mock were injected into the peritoneal cavity of mice. After 24 hours, tissue samples were obtained from the liver, spleen, lymph nodes, lung, and kidney. The localization of polyplex micelles was then examined under a laser scanning confocal microscope (A1+, Nikon Instruments, Tokyo, Japan).

### Validation of transgene expression after administration of polyplex micelles

For *in vitro* experiments, human SUIT2 cancer cells were treated with PEG-*b*-P[Asp(DET)]/P[Asp(DET)] mixed micelles encapsulating SART3, CD40L, and GM-CSF genes for 48 hours. For *in vivo* experiments, PEG-*b*-P[Asp(DET)]/P[Asp(DET)] mixed micelles encapsulating the GM-CSF gene were injected into the peritoneal cavity of mice. The liver, spleen, lung, kidney, and lymph nodes of the mice were obtained after 24 hours. The expression levels of the transgenes were determined by real-time RT-PCR.

### Real-time RT-PCR

Total RNA was extracted using an Illustra RNAspin Mini RNA Isolation Kit (GE Healthcare, Uppsala, Sweden). cDNA was synthesized using a Transcription First Strand cDNA synthesis Kit (Roche Applied Science). Real-time RT-PCR was performed with a LightCycler480 II (Roche Diagnostics) (n = 4) using the fluorescence-labeled locked nucleic acid-probe and primer sets as follows: FAM-ctggctgg-quencher; 5′-GTGAGCTCTTCCCCCTGAC-3′ and 5′-CATGCTGATCTCATCGTGGA-3′for mouse SART3, FAM-cctcctgg-quencher; 5′-GGCCTTGGAAGCATGTAGAA-3′ and 5′-TCTGCACATGTTAGCTTCTTG-3′for mouse GM-CSF, FAM-cagcatcc-quencher; 5′-ACGTTGTAAGCGAAGCCAAC-3′ and 5′-TATCCTTTCTTGGCCCACTG-3′ for mouse CD40L, and the Universal ProbeLibrary ACTB Gene Assay for β-actin (Roch Applied Science).

### Mouse tumor models and vaccination protocols

In preliminary experiments, we confirmed overexpression of SART3 protein in CT26 and 3LL/LLC cancer cells but weak protein expression of SART3 in normal organ tissues (spleen, lymph node, liver, kidney, and lung) by western blot analyses (Fig. S1), and conducted vaccinations of two mouse strains and various tumor models. At the time of organ/tissue sampling and termination of survival monitoring, the mice were euthanized by cervical dislocation under anesthesia induced by isoflurane inhalation.

Peritoneal dissemination model of cancer: Syngeneic CT26 colon cancer cells were injected into the peritoneal cavity of BALB/c mice (1×10^5^ cells/mouse; day 0). Then, polyplex micelles encapsulating the indicated genes (Table 1) were intraperitoneally administered four times at 1 week intervals (days 1, 8, 15, and 22). Mouse survival was monitored every day until day 80 after the first challenge with CT26 cells. Mice that survived for more than 80 days were subcutaneously injected with CT26 cells (1×10^6^ cells/mouse) in the flank region (re-challenge experiment). The formation of subcutaneous tumors was monitored for the following 60 days. In some experiments, splenocytes were isolated from long-term surviving mice and subjected to the CTL and natural killer (NK) cell assays.

**Table 1:** Therapeutic genes encapsulated by polyplex micelles and the survival periods of mice with peritoneal dissemination of CT26 tumors.

	Median survival (days)
Mock (50 µg) (n = 19)	32.0
SART3 (25 µg) + Mock (25 µg) (n = 6)	37.0
CD40L (25 µg) + Mock (25 µg) (n = 8)	38.5
GM-CSF (25 µg) + Mock (25 µg) (n = 10)	46.0**
SART3 (25 µg) + CD40L (25 µg) (n = 10)	34.5
SART3 (25 µg) + GM-CSF (25 µg) (n = 7)	47.0*
SART3/CD40L (25 µg) + GM-CSF (25 µg) (n = 18)	77.0^†^

SART3: squamous cell carcinoma antigen recognized by T cells 3; GM-CSF: granulocyte macrophage colony-stimulating factor. *P<0.05, **P<0.01, and ^†^ *P*<0.0001 vs. Mock control.

Subcutaneous tumor model: Syngeneic CT26 colon cancer or LLC lung cancer cells were subcutaneously injected into the flank region of BALB/c or C57BL/6 mice (1×10^6^ cells/mouse; day 0), and then polyplex micelles with therapeutic genes were intraperitoneally administered two or four times, respectively, at 1 week intervals (days 1, 8, 15, and 22). In BALB/c mice, subcutaneous CT26 tumors were obtained and the tumor weight was measured on day 14 to compare the therapeutic efficacies of the groups. In C57BL/6 mice, subcutaneous LLC tumors, spleen, and lymph nodes were obtained on day 28 to examine tumor weights, the presence of lung metastasis, and infiltration of immune cells in these tissues. In some experiments, splenocytes were freshly isolated and subjected to CTL and NK cell assays.

### CTL and NK cell assays

Freshly isolated splenocytes (5×10^7^) were co-cultured with 20 Gy-irradiated CT26 or LLC/3LL cells (5×10^6^) in 20 ml RPMI 1640 medium supplemented with 10% FBS, 5×10^−5^ M 2-mercaptoethanol, 100 U/ml penicillin, and 100 µg/ml streptomycin at 37°C in a humidified incubator with 5% CO_2_. After 72 hours of incubation, the splenocytes were harvested and used as effector cells for CTL and NK cell assays as described previously [24]. CT26 or 3LL/LLC target cells for CTL assays and YAC-1 target cells for NK cells assays were resuspended with RPMI 1640 medium at a density of 20×10^6^ cells/ml and labeled with 10 µM 6-(*N*-Succinimidyloxycarbonyl)-fluorescein 3′, 6′-diacetate (CFSE) (Dojindo Laboratories, Kumamoto, Japan) for 10 minutes at 37°C. The reaction was stopped by addition of an equal volume of FBS. After two washes with RPMI 1640 medium, CFSE-labeled target cells were immediately combined with the effector cells at target/effector (T/E) ratios of 1/0, 1/25, 1/50, or 1/100 (T: 1×10^4^ cells/E: 0, 25×10^4^, 50×10^4^, or 100×10^4^ cells, respectively) in 200 µl RPMI 1640 medium and incubated for 6 hours. Flow-Count Fluorospheres (10,000 in each sample, Coulter Corporation) and propidium iodide (1 µg/ml, stains dead cells) were added to the co-cultures just prior to analysis by flow cytometry (FACSCanto II; BD Bioscience, Mountain View, CA). To facilitate counting the number of target cells, 2,000 events for the Fluoroshpere-microbeads were collected and used as a reference in CellQuest software (BD Bioscience). The percentage of live cells was calculated as follows: [number of viable CFSE^+^ target cells at T/E ratios of 1/25–1/100]/[number of viable CFSE^+^ target cells at a T/E ratio of 1/0] ×100.

### MHC-blocking and SART3-knockdown experiments

Blocking experiments were performed to analyze the MHC restriction of target cell lysis in CTL assays. CT26 target cells were incubated with saturated concentrations of anti-MHC class I or isotype control antibodies (H-2L^d^: 28-14-8, BioLegend, San Diego, CA and H-2K^d^: SF1-1.1.1, eBioScience, San Diego, CA, respectively) for 30 minutes before adding to effector cells. After 48 hours, the treated CT26 cells were co-cultured with effector cells. Independent experiments were repeated twice.

SART3 expression in CT26 target cells was knocked down by transfection of siRNA (50 nM; sense, 5′-CUACAGUCAGUACCUAGAUdTdT-3′ and antisense, 5′-AUCUAGGUACUGACUGUAGdTdT-3′) using Lipofectamine 2000 (Life Technology). After 48 hours, the SART3 knockdown CT26 cells were co-cultured with effector cells.

### Immunohistochemistry

Tissue samples were sectioned and fixed in ice-cold acetone for 10 minutes. The sections were incubated with 3% H_2_O_2_ and 1% bovine serum albumin. The specimens were then incubated with primary antibodies against CD4 (1∶250. #100505, BioLegend), CD8a (1∶1000, #100701, BioLegend), CD11c (1∶500, ab33483, Abcam, Cambridge, UK), or GM-CSF (1∶1000, ab13789, Abcam) at room temperature for 1 hour. Then, the stained samples were developed with 3, 3′-diaminobenzidine using the Vectastain biotin/avidin system (Vector Labs, Burlingame, CA), followed by hematoxylin and eosin counterstaining. Immunostaining was quantitated by optical microscopy (ECLIPSE 55i, Nikon Instruments) using the NIS-Elements D 3.2 quantitative analysis program.

### Flow cytometric analysis

Splenocytes were isolated at 48 hours after the last administration of polyplex micelles to subcutaneous tumor models. The cells were then subjected to flow cytometric analysis using PE-conjugated anti-CD11c (Miltenyi Biotec Gmbh, Bergisch Gladbach, Germany), FITC-conjugated anti-CD11b (Abcam), anti-CD80, anti-CD86 (Beckman Coulter, Fullerton, CA), and anti-MHC-Class II (Miltenyi Biotec Gmbh) monoclonal antibodies.

### In vivo CD4^+^ and CD8^+^ T cell depletion

To examine the role of CD4^+^ and CD8a^+^ T cells in antitumor immunity against CT26 tumors, BALB/c mice were depleted of either CD4^+^ or CD8a^+^ T cells. At 3 days before DNA vaccinations, mice were injected four times with anti-CD4^+^ or -CD8a^+^ subset-specific monoclonal antibodies (GK1.5 and 53–6.7, respectively; BioLegend), or isotype rat IgG (RTK4530; BioLegend) as a control (150 µg per mouse). Survival rates were then compared among the groups.

### Statistical analysis

Results are represented as means ± standard deviation. The differences between two or more than three groups were statistically analyzed using the Student's *t*-test or one-way analysis of variance followed by Dunn's test, respectively. Survival curves were evaluated by the Kaplan-Meier method and analyzed with the log-lank test. *P-*values of less than 0.05 were considered significant.

## Results

### Preparation of polyplex micelles

The BH polyplex micelles were prepared according to the procedure determined previously [16]. In brief, the polymer solution including B and H at the previously optimized B/H composition of 70/30 was mixed with pDNA solution to form polyplex micelles. The polyplex micelles were characterized to have a neutral ζ-potential of 1.55±1.16 mV (n = 3) and cumulant diameter of 91.3±3.2 nm with a unimodal size distribution (PDI 0.16±0.02, n = 3) from DLS measurement (Fig. S2), which agreed well to our previous report [16].

### Tissue localization of polyplex micelles and transgene expression

**Figure 1:**
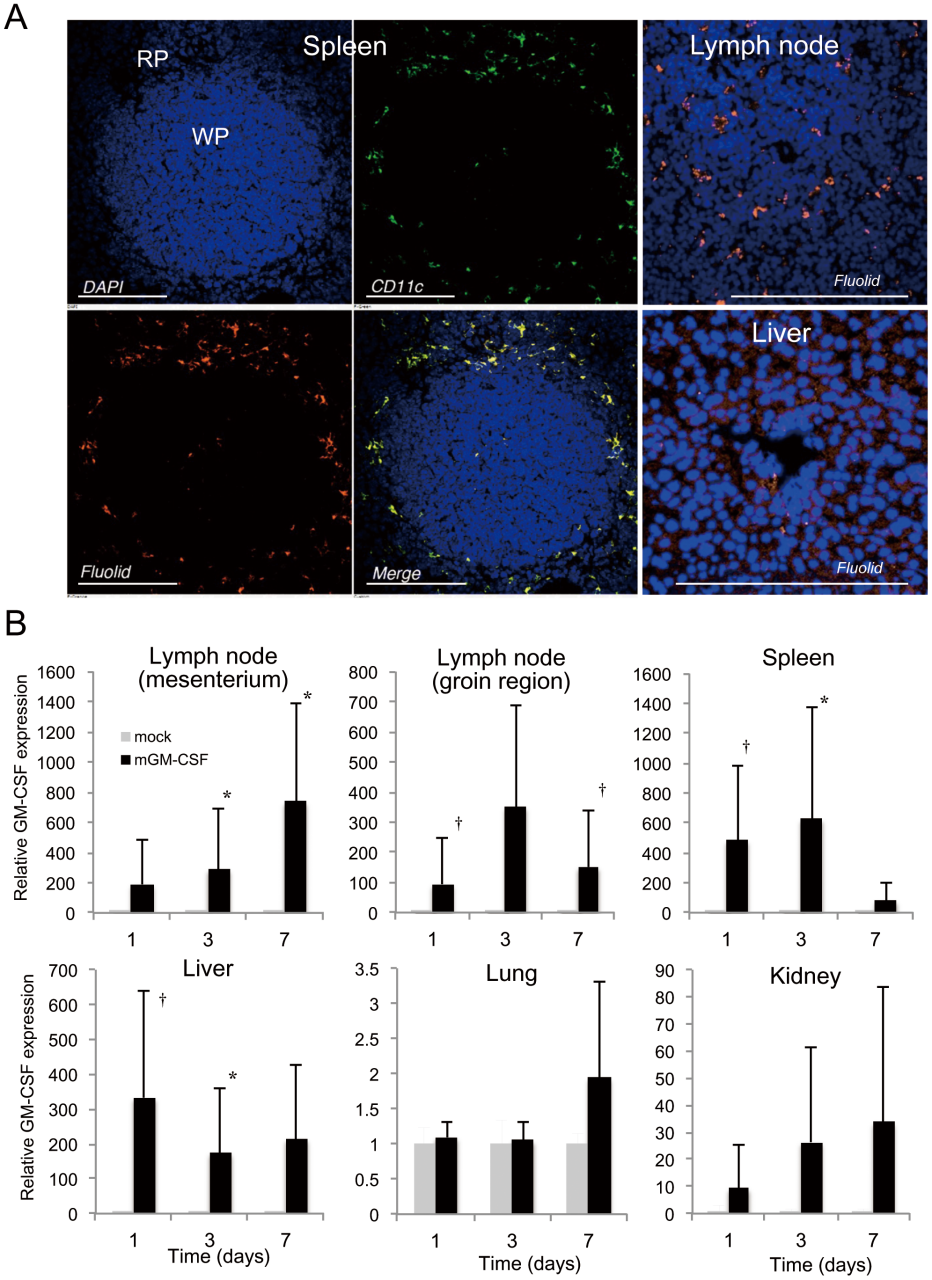
Polyplex micelle distribution and transgene expression *in vivo*. Fluolid orange-labeled polyplex micelles with the GM-CSF gene (50 µg; N/P ratio  = 10) were administered to the peritoneal cavity of mice. (A) Polyplex micelles were mainly localized in the spleen, lymph nodes, and liver. The merged image (yellow) shows co-localization of polyplex micelles (orange) and CD11c^+^ DCs (green). DAPI nuclear staining (blue). RP: red pulp of spleen; WP: white pulp of spleen. (B) Total RNA was extracted from frozen tissues after i.p. administration, followed by real-time RT-PCR analysis of GM-CSF gene expression. The gene expression of GM-CSF was up-regulated by several-hundred fold compared with that in the mock control (relative expression  = 1) in lymph nodes, spleen, and liver, and minimally in the lungs and kidney (n = 4 each). *P<0.05; †*P*<0.001. Scale Bar  = 200 µm.

At 24 hours after i.p. administration, fluolid orange-labeled polyplex micelles containing GM-CSF pDNA (50 µg) (NP ratio of 10) were mainly localized in the spleen, mesenteric lymph nodes, and liver (Fig. 1A). To analyze sub-localization of polyplex micelles among the spleen cells, frozen spleen sections were immunostained for CD11c. Fluolid signals (orange) were well co-localized with FITC-CD11c signals (green) (Fig. 1A, left panel, yellow). Flow cytometric analysis showed that the percentages of Fluolid^+^/CD11c^+^ and Fluolid^+^/CD11b^+^ cells among Fluolid^+^ splenocytes were 44.4% and 3.5%, respectively, suggesting that polyplex micelles were predominantly distributed in DCs and to a lesser extent in macrophages.

In a preliminary experiment, we confirmed that incubation of human SUIT2 cancer cells with mouse SART3/CD40L/GM-CSF gene-loaded polyplex micelles resulted in expression of all transgenes *in vitro* (Fig. S3). At 24 hours after i.p. administration of polyplex micelles, *in vivo* gene expression levels of GM-CSF in lymph nodes, spleen, and liver (n = 4; Fig. 1B) were several hundred-fold higher than those in mock controls. GM-CSF expression was sustained at high levels for 1 week post-administration of polyplex micelles. In contrast, upregulation of GM-CSF expression was much less evident in the kidney and lung (n = 4 each) compared with that in the lymphatic tissues.

### Polyplex micelle-based DNA vaccination with SART3, CD40L, and GM-CSF genes prolongs the survival of mice harboring peritoneal dissemination of cancer cells

**Figure 2:**
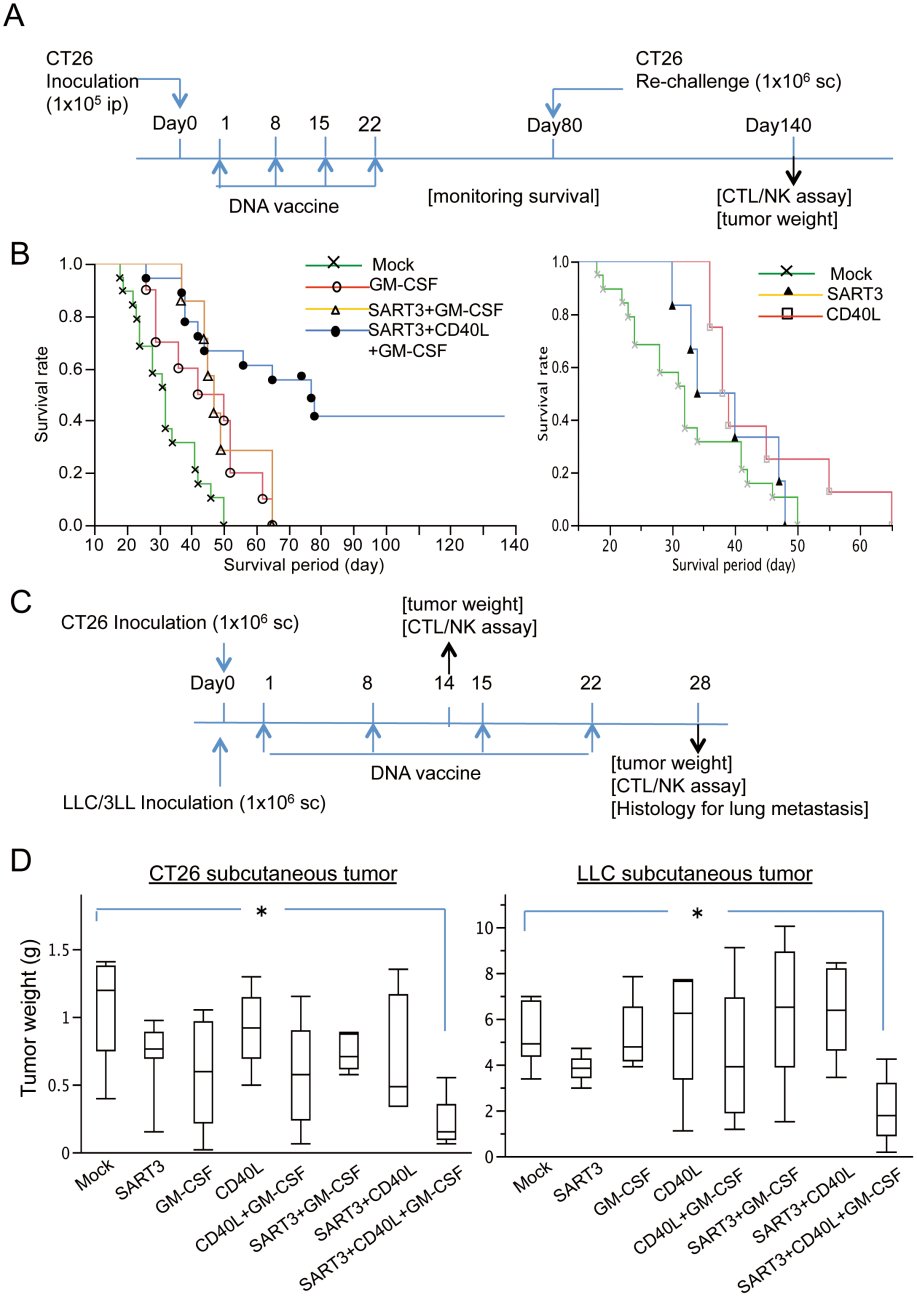
Anti-tumor efficacy of the polyplex micelle-based DNA vaccine in mice harboring peritoneal and subcutaneous tumors. (A) Vaccination schedule of polyplex micelles encapsulating therapeutic genes (Table 1) in the peritoneal dissemination model of CT26 tumors. (B) There were significant increases in the survival rates of SART3/CD40L+GM-CSF, GM-CSF, and SART3+GM-CSF groups (*P*<0.0001, n = 18; *P*<0.01, n = 10; *P*<0.01, n = 7 vs. mock control, n = 19, respectively; left panel). Long-term surviving mice were only obtained by vaccination of polyplex micelles with SART3, CD40L, and GM-CSF genes. No significant improvement in survival was detected by transfection of SART3 or CD40L genes alone (n = 6 and 10; right panel). (C) Vaccination schedule of polyplex micelles with therapeutic genes in subcutaneous CT26 and LLC tumor models. (D) Polyplex micelles with SART3, CD40L, and GM-CSF genes (n = 7 and 9), but not other transgenes (n = 5−6), significantly decreased the weights of CT26 (left panel) and LLC subcutaneous tumors (right panel) compared with those in the mock control (n = 6 and 7). *P<0.01.

Following the protocol described in Figure 2A, polyplex micelles were administered to mice harboring peritoneal dissemination of CT26 cells. The survival periods for groups that received the indicated transgenes are summarized in Table 1 (n = 6−19 per group). Polyplex micelles encapsulating genes encoding SART3, CD40L, CD40L+GM-CSF, or SART3+CD40L did not significantly prolong survival compared with that in the mock control (median survival: 37, 38.5, 32, and 34.5 days vs. 32 days, respectively). On the other hand, polyplex micelles encapsulating genes encoding GM-CSF or SART3+GM-CSF improved survival (46 and 47 days, respectively) compared with that in the control. Polyplex micelles with SART3, CD40L, and GM-CSF genes resulted in the longest survival at 77 days. The survival rates (Fig. 2B, left panel) of SART3/CD40L+GM-CSF, GM-CSF, and SART3+GM-CSF groups (*P*<0.0001, *P<*0.01, and *P<*0.05, respectively) were higher than those of the control. On the other hand, survival rates were not improved by polyplex micelles with SART3, CD40L, CD40L+GM-CSF, or SART3+CD40L genes (Fig. 2B, right panel), or naked plasmids of SART3/CD40L+GM-CSF without polyplex micelles (data not shown). Notably, only SART3/CD40L+GM-CSF gene-loaded polyplex micelles resulted in long-term survival (macroscopic cure) of 40% of the vaccine-recipient mice.

### Polyplex micelle-based DNA vaccination with SART3, CD40L, and GM-CSF genes inhibits the growth of subcutaneous tumors

After i.p. administration of the polyplex micelles according to the protocol in Figure 2C, the SART3/CD40L+GM-CSF gene-loaded DNA vaccine significantly decreased the growth of subcutaneous CT26 tumors compared with that in the mock control (0.22±0.17 g, n = 7 vs. 1.1±0.39 g, n = 6; *P*<0.01). Tumor growth inhibition in groups (n = 5−7 in each group) treated with CD40L (0.92±0.28 g), SART3 (0.72±0.27 g), GM-CSF (0.60±0.40 g), CD40L+GM-CSF (0.69±0.49 g), SART3+GM-CSF (0.74±0.13 g), or SART3+CD40L (0.69±0.44 g) did not reach statistical significance compared with that in the mock control (Fig. 2D: left panel).

To validate the efficacy of the DNA vaccine for different MHC haplotypes and tumor types, we used subcutaneous LLC/3LL tumors in CB57/BL6 mice, which have a different MHC class 1 haplotype, H-2b. As shown in Figure 2D (right panel), the growth of LLC tumors was significantly suppressed in the SART3/CD40L+GM-CSF gene-loaded DNA vaccine group (2.0±1.4 g, n = 9, *P<*0.01) compared with that in the mock control (5.1±1.3 g, n = 7). In contrast, other treatments (n = 5−6 in each group) with SART3 (3.9±0.6 g), GM-CSF (5.3±1.5 g), CD40L (5.7±2.7 g), CD40L+GM-CSF (4.3±3.5 g), SART3+GM-CSF (6.5±3.5 g), or SART3+CD40L (6.4±2.0 g) did not inhibit tumor growth.

### Polyplex micelle-based DNA vaccination with SART3, CD40L, and GM-CSF genes inhibits lung metastasis of subcutaneous LLC tumors

**Figure 3:**
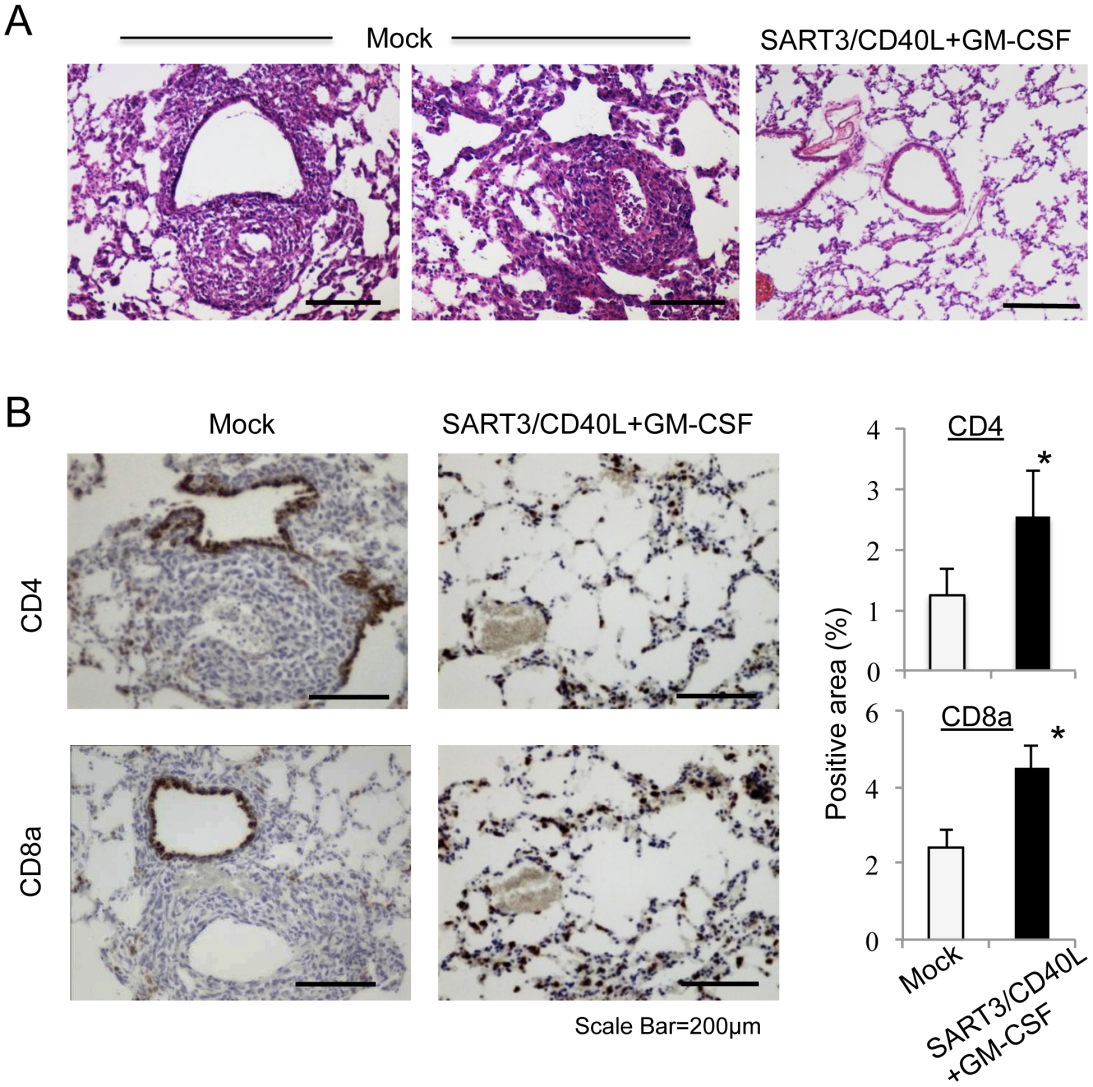
Protective effect of the polyplex micelle-based DNA vaccine on lung metastasis of LLC tumors. (A) Hematoxylin and eosin staining of lung tissues showed that lung metastatic nodules were highly developed by day 28 after subcutaneous injection of LLC cancer cells in the mock control (4/4 cases), but not present in the SART3/CD40L+GM-CSF vaccine group (0/4 cases). (B) Lung tissues were immunostained with anti-CD4 or CD8a antibodies. Increased infiltration of CD4^+^ and CD8a^+^ T cells into the lung beds was observed in the SART3/CD40L+GM-CSF group. *P<0.05, n = 4.

Because LLC/3LL cancer is known to exhibit a highly metastatic potential, we monitored the occurrence of lung metastasis for 28 days in the aforementioned mice harboring subcutaneous LLC tumors. Histological examination detected lung metastases in 100% (4/4 cases) of mice in the mock control (Fig. 3A, left panel). In contrast, mice administered the SART3/CD40+GM-CSF gene-loaded DNA vaccine showed no development of lung metastasis (0/4 cases; Fig. 3A, right panel) and greater regression of tumor sizes (Fig. 2D, right panel). Many immune cells had infiltrated into the lung beds of mice that received the SART3/CD40L+GM-CSF gene-loaded vaccine. Therefore, we examined these lung tissues by immunohistochemistry (Fig. 3A), and found that the number of infiltrated CD4^+^ and CD8a^+^ immune cells was increased by two-fold compared with that in the mock control (*P*<0.05 and *P*<0.01, respectively, n = 4 each; Fig. 3B). Bronchial epithelia that immunoreacted with the anti-CD4 and CD8a antibodies were excluded from the quantitation.

### CTL and NK cell cytotoxicities are enhanced by the polyplex micelle-based DNA vaccine with SART3, CD40L, and GM-CSF genes

**Figure 4:**
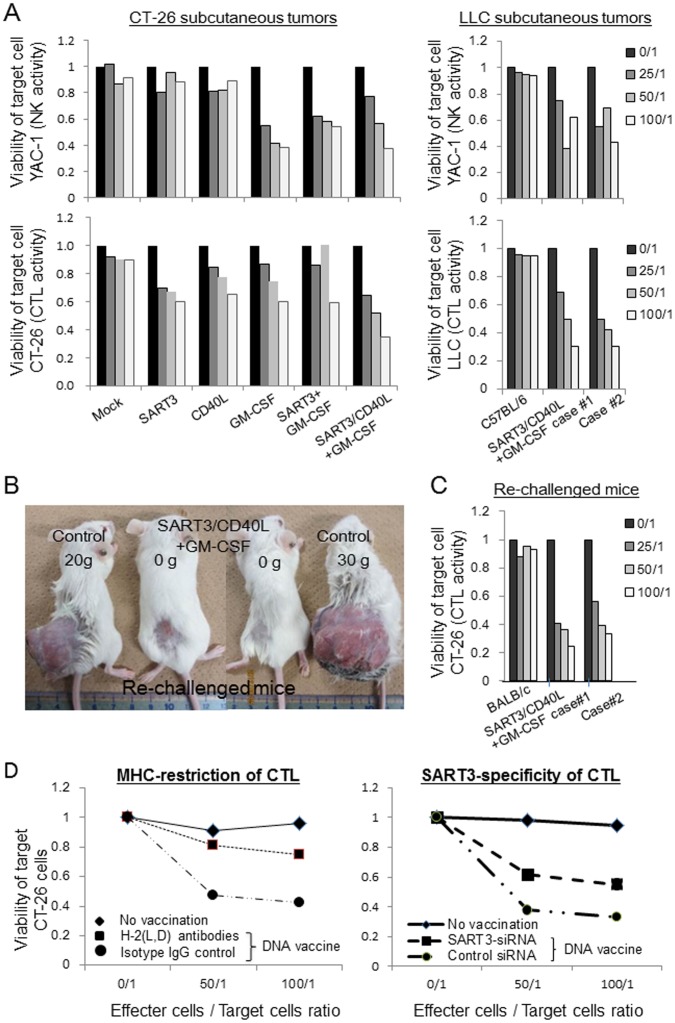
Polyplex micelle-based DNA vaccine induces CTL activation and memory immunity. (A) Splenocytes (effector cells) were isolated from mice bearing CT26 and LLC subcutaneous tumors, and then co-cultured with irradiated CSFE-labeled CT26 or YAC-1 target cells at the indicated E/T cell ratios. NK cell activity (upper panel) was increased in all treatment groups with the GM-CSF transgene. In contrast, CTL activity (lower panel) was remarkably elevated by the polyplex micelle encapsulating SART3/CD40L+GM-CSF genes (DNA vaccine group) in an E/T cell ratio-dependent manner. (B) CT26 cells were re-challenged in the flank region of mice that survived for more than 80 days. The formation of subcutaneous tumors was monitored for a further 60 days. Complete rejection of re-challenged tumor cells was detected in the SART3/CD40L+GM-CSF vaccine group, but not in the control. (C) Splenocytes isolated from mice with re-challenge of CT26 cells were subjected to the CTL assay. CTL activity was increased in long-term surviving mice that received the SART3/CD40L+GM-CSF vaccine, but not in the control. (D) CTL activity against CT26 target cells with anti-MHC class 1 (H-2L and -2D) antibodies in mice that were administered the SART3/CD40L+GM-CSF vaccine was reduced to the almost same level as that in the no vaccination control (left panel; n = 2). The CTL activity against SART3-knockdown CT26 cells in mice that were administered the SART3/CD40L+GM-CSF vaccine was reduced compared with that against control CT26 cells, but the knockdown efficiency of SART3 siRNA at the protein level was only 50% (right panel; n = 2).

First, we examined cytotoxic NK cell activity because activation of innate immunity is a prerequisite for induction of acquired immunity. None of the polyplex micelles encapsulating Mock, SART3, or CD40L pDNA increased NK cell activity (Fig. 4A, upper panel). However, administration of polyplex micelles containing the GM-CSF transgene, including GM-CSF, SART3+GM-CSF, and SART3/CD40L+GM-CSF, upregulated NK cell activity in both CT26 and LLC subcutaneous tumor models.

To evaluate CTL activity, we employed a CFSE-based cytotoxicity assay using CT26 or LLC cells as target cells because of its high sensitivity [25]. In the CT26 subcutaneous tumor model (Fig. 4A, left bottom panel), the number of viable CT26 target cells was decreased by SART3/CD40L+GM-CSF gene-loaded polyplex micelles, but not mock control, GM-CSF- or SART3+GM-CSF gene-loaded polyplex micelles. In the LLC subcutaneous tumor model (Fig. 4A, right bottom panel), the number of viable LLC target cells was also decreased by the SART3/CD40L+GM-CSF vaccine in an E/T cell ratio-dependent manner (Fig. 4A, right bottom panel). BALB/c mice have MHC haplotype “d”, whereas C57BL/6 mice have haplotype “b”. These results indicate an advantage of our DNA vaccine is that identification of effective epitopes of the TAA protein for matching with MHC haplotypes is unnecessary.

### Tumor re-challenge verifies acquired rejection immunity in mice treated with the DNA vaccine

Among mice with peritoneal dissemination of CT26 cells, long-term surviving mice were observed only in the group that received the SART3/CD40L+GM-CSF DNA vaccine. To elucidate whether the DNA vaccine elicited memory immunity with CT26 cell-specific rejection, re-challenge with 1×10^6^ CT26 cells was performed in long-term survivors in comparison with non-vaccinated controls. As shown in Figure 4B, the re-challenged CT26 tumor was completely rejected in the DNA vaccine group (100%, 8/8), but subcutaneous tumors had developed in control mice. The NK cell activity (data not shown) and CTL activity (Fig. 4C) were increased in mice that received the DNA vaccine regimen in an E/T cell ratio-dependent manner. In contrast, CTL and NK cell activity was not elevated in control mice (Fig. 4C).

### MHC- and SART3-specific CTL killing activity

We verified the MHC restriction of CTL activity using MHC (H-2L and H-2D)-blocking antibodies (Fig. 4D, left panel). The CTL activity of splenocytes from mice that received the DNA vaccine with SART3, CD40L, and GM-CSF genes under MHC-blocking conditions was remarkably reduced to one-third of the control treated with the DNA vaccine and isotype control antibody.

Although we tried to knock down SART3 expression in CT26 cells using SART3-targeted siRNA, the level of protein expression was 50% of that in the control. The CTL activity against the SART3-knockdown CT26 cells was reduced, but the degree was not as high as that obtained by treatment with MHC-blocking antibodies (Fig. 4D, right panel).

### Infiltrated CD11c^+^ cells in lymph nodes, spleen, and tumors are increased and activated by DNA vaccine treatment

**Figure 5:**
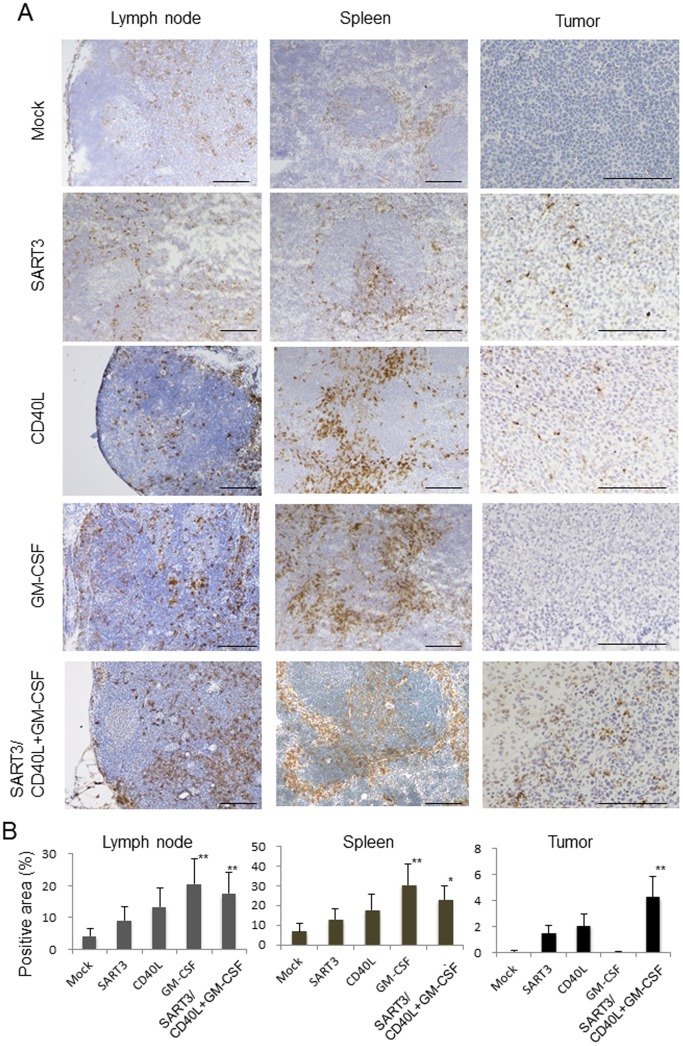
Immunohistochemical analysis of CD11c^+^ DCs in lymph node, spleen, and tumor tissues. (A) Tissue sections of the lymph nodes, spleen, and tumors from mice that received polyplex micelles with the indicated transgenes were immunostained with an anti-CD11c antibody. Scale Bar  = 200 µm. (B) The number of CD11c^+^ DCs was significantly increased in the lymphatic organs of GM-CSF and SART3/CD40L+GM-CSF transfection groups. In tumor tissues, a significant increase of CD11c^+^ DC numbers was detected in SART3/CD40L+GM-CSF vaccine group. *P<0.05, **P<0.01 vs. mock control (n = 5).

Immunohistochemical analysis revealed an increased number of CD11c^+^ cells in the lymph nodes and spleen (Fig. 5A, left and middle panels) of mice administered polyplex micelles with GM-CSF or SART3/CD40L+GM-CSF genes compared with that in the control (*P<*0.05, n = 4; Fig. 5B). However, the number of CD11c^+^ cells in the lymph nodes and spleen was not remarkably changed in mice administered polyplex micelles with CD40L or SART3 transgenes. In tumor tissues, CD11c^+^ cell infiltration was significantly increased by administration of SART3/CD40L+GM-CSF gene-loaded polyplex micelles (Fig. 5A, right panel; *P<*0.05 vs. mock control, n = 4; Fig. 4B), but no infiltration of CD11c^+^ cells was detected in the GM-CSF transgene group.

To examine the maturation of CD11c^+^ DCs, we analyzed the expression levels of CD80, CD86, and MHC class II on isolated CD11c+ splenocytes by flow cytometry. Polyplex micelles with GM-CSF or CD40L/GM-CSF genes slightly increased the number of CD11c^+^/CD80^+^ and CD11c^+^/CD86^+^ double positive cells (*P<*0.05) compared with that in the mock control. On the other hand, polyplex micelles with SART3/CD40L/GM-CSF genes increased the numbers of CD11c^+^/MHC class II^+^ cells (*P<*0.05) and CD11c^+^/CD80^+^ and CD11c^+^/CD86^+^ double positive cells more significantly (*P<*0.01) (Fig. S4).

### Infiltration of CD4^+^ and CD8a^+^ T cells into tumors is increased by DNA vaccine treatment

**Figure 6:**
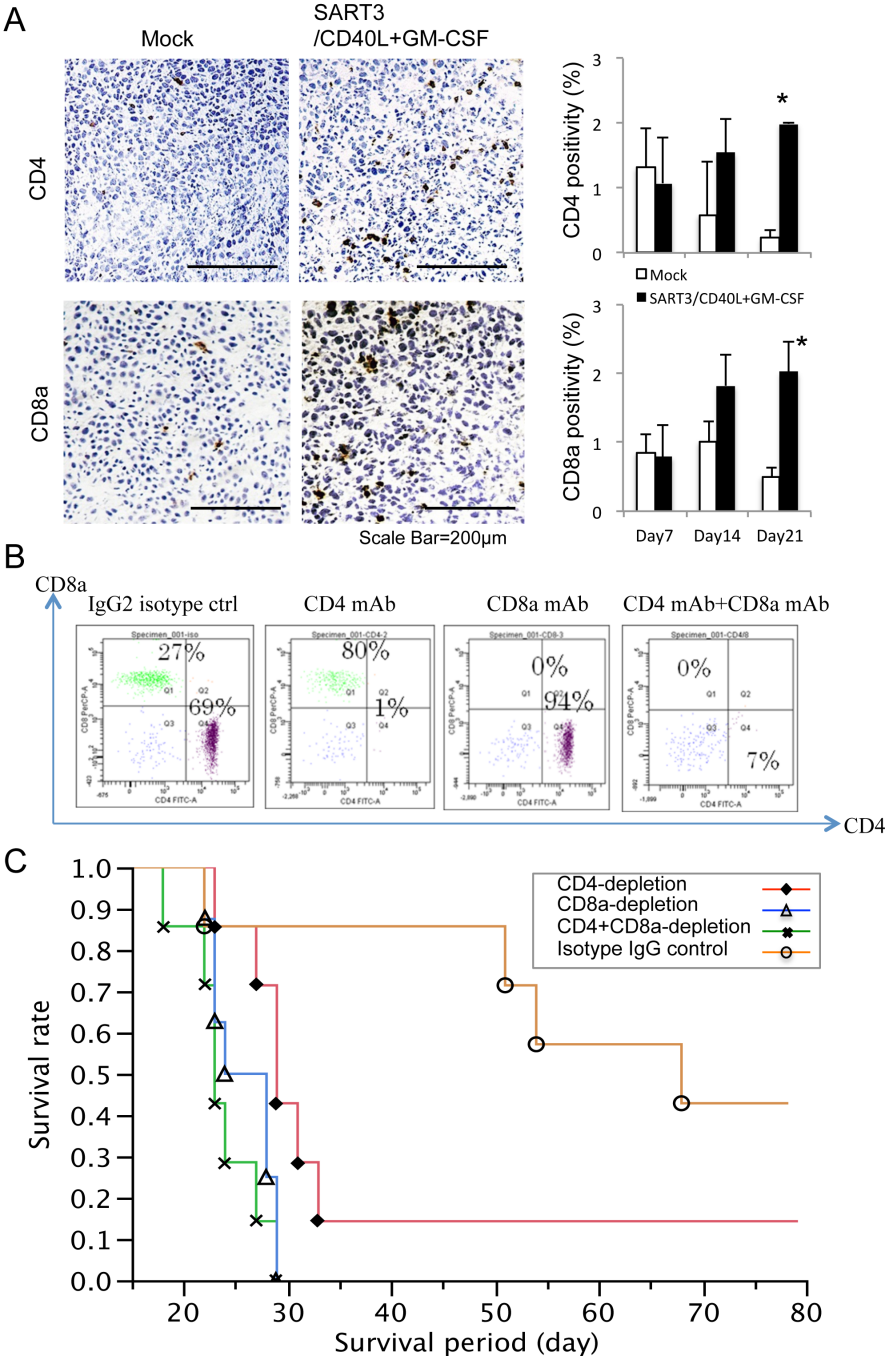
CD4^+^/CD8a^+^ cell numbers increase in tumors by SART3/CD40L+GM-CSF vaccination and depletion deteriorates the anti-tumor efficacy. (A) Tissue sections of subcutaneous CT26 tumors after administration of polyplex micelles were immunostained with anti-CD4 or CD8a antibodies. CD4^+^ and CD8a^+^ T cells were highly infiltrated into tumors of the SART3/CD40L+GM-CSF vaccine group compared with that in the mock control. *P<0.05 (n = 4). (B) Depletion of CD4^+^ and/or CD8a^+^ T cells by i.p. administration of their neutralizing antibodies was confirmed by flow cytometric analysis of blood samples. (C) Kaplan-Meier analysis showed that depletion of CD4^+^ or CD8a^+^ T cells decreased the survival of the SART3/CD40L+GM-CSF vaccine group compared with that that of the isotype IgG2 control (*P* = 0.084 and 0.003, respectively, n = 7−8) in mice with peritoneal dissemination of CT26 cells.

We examined the infiltration of CD4^+^/CD8a^+^ T cells into tumor tissues after administration of SART3/CD40L+GM-CSF gene-loaded polyplex micelles by immunohistochemical analysis (Fig. 6A). The number of CD4^+^/CD8a^+^ T cells was significantly increased by the SART3/CD40L+GM-CSF vaccine compared with that in the mock control on days 14 and 21 (*P*<0.05 on day 21, n = 4).

### Depletion of CD4^+^ and CD8a^+^ T cells in vivo reduces the anti-tumor effect of the DNA vaccine in mice with peritoneal dissemination of CT26 cells

Flow cytometric analysis (Fig. 6B) showed that administration of anti-CD4 and -CD8a antibodies depleted almost all CD4^+^ and CD8a^+^ cells in blood samples of mice, after which they received the DNA vaccine containing SART3, CD40L, and GM-CSF genes. CD4- or CD8a-depleting antibodies shortened the median survival of the vaccinated mice compared with that of the isotype IgG control (29 and 26 days vs. 68 days, n = 7−8; *P* = 0.084 and *P*<0.01, respectively; Fig. 6C). Compared with CD4^+^ cell depletion, double depletion of CD4^+^/CD8a^+^ cells further shortened the survival (23 days, n = 7) with a marginal significance (*P*<0.05), but there was no difference in CD8a^+^ and CD4^+^/CD8a^+^ cell depletion groups (*P* = 0.31).

## Discussion

In the present study, we developed a novel DNA vaccine platform using a non-viral synthetic gene carrier, block/homo-mixed polyplex micelles. In peritoneal dissemination and subcutaneous tumor models, the SART3/CD40L+GM-CSF-loaded polyplex micelles were predominantly distributed to DCs in the lymph nodes, spleen, and liver after i.p. administration, inhibited the growth and metastasis of subcutaneous tumors, and prolonged the survival of mice with peritoneal dissemination of cancer cells. The anti-tumor effect was elicited by DC activation and then the infiltration of CD4^+^ (helper) and CD8a^+^ (cytotoxic) T cells into tumors. This mechanism was supported by the fact that *in vivo* CD4^+^ or CD8a^+^ cell depletion reversed the anti-tumor efficacy of the vaccine. This is the first report of a vaccination effect in which DC as well as helper and cytotoxic T cell activation is achieved by i.p. administration of polyplex micelles with SART3, CD40L, and GM-CSF genes.

Nano-sized gene carriers have the characteristics to be absorbed into lymphatic drainage routes following i.p. administration [26]. For example, gene delivery in mannose-modified ultrasound-responsive bubble liposomes promotes high expression of transgenes in lymphatic organs by ultrasound exposure, suggesting their potential application in DNA vaccination [27]. Our block/homo polyplex micelles were also predominantly distributed in lymphatic organs (lymph nodes and spleen) by i.p. administration and delivered genes without additional induction methods. The distribution of polyplex micelles in our mouse model without large tumors is consistent with our previous study in mice harboring macroscopic dissemination of cancer cells [14], [15]. Furthermore, we analyzed the sub-localization in these organs and found that polyplex micelles delivered the transgenes to APCs, predominantly DCs, and macrophages to a lesser extent and induced their activation. Polyplex micelles of around 100 nm have appropriate characteristics as a gene delivery platform for DNA vaccination by i.p. administration.

SART3 was identified as a TAA by a cDNA expression cloning method using cancer-reactive tumor-infiltrating lymphocytes [20]. Here, we tested SART3 as a model TAA gene because SART3 is an autologous TAA for mice [23], and several sequences of peptides can be presented by different human and mouse haplotypes of HLA/MHC molecules [21], [22]. To induce immune responses against weakly immunogenic TAAs, complete and/or incomplete Freund's adjuvants are co-injected with peptide vaccines [28]. For cell vaccines, viral and bacterial pCpG motifs may work as adjuvants [29], and DCs have a high potential for antigen presentation [1]. For genetic vaccines, adjuvant molecules, such as polyubiquitin-fusion sequences [30] and heat-shock proteins for a scavenger [31], have been studied to resolve the issues of weak immunogenicity. In this study, we tried a simple and versatile approach by co-expression of CD40L and GM-CSF together with a TAA gene using non-viral polyplex micelle-based gene carriers, because tumor cell/DC-based vaccines with GM-CSF or CD40L work as a high potential vaccine [32], [33]. Transfection of the SART3 gene alone neither upregulated CTL activity nor induced complete tumor rejection. In contrast, the combination of SART3, CD40L, and GM-CSF genes resulted in a macroscopic cure (40% of all recipients) and protected against distant metastasis in all mice through high CTL activity. These results suggest that TAA gene transfection is insufficient and simultaneous expression of CD40L and GM-CSF is necessary to elicit a strong vaccine effect against weakly immunogenic tumors.

We found differences in the number and distribution of CD11c^+^ DCs by gene transfection of SART3, CD40L, or GM-CSF genes alone and their combination in immunohistochemistry of lymphatic organs and tumor tissues. Transfection of the GM-CSF gene increased the number of DCs in the lymph nodes and spleen, but did not stimulate the infiltration of DCs into tumor tissues. In contrast, SART3 gene transfection did not increase the number of DCs in lymphatic organs, while a small number of DCs had infiltrated into tumor tissues. On the other hand, transfection of the CD40L gene stimulated the infiltration of DCs into lymphatic organs to a much lesser degree than that by GM-CSF gene transfection. In tumor tissues, DC infiltration was slightly induced by CD40L gene transfection, although the underlying mechanism remains unclear. Because CD40L is known to enhance DC maturation [33], the increase and activation of DCs with upregulation of CD80, CD86, and MHC class II may only occur when both GM-CSF and CD40L stimulate DCs together with TAA gene vaccination.

Transfection of the GM-CSF gene may activate not only DCs but also NK cells, because treatment with GM-CSF gene-loaded polyplex micelles increased NK cell activity. The therapeutic efficacy of GM-CSF gene transfection alone was different in the two types of tumor models. The survival period was slightly extended for mice harboring peritoneal dissemination of cancer cells, but there was no growth inhibition of subcutaneous tumors. Small-sized disseminated nodules may be directly contacted and suppressed by activated NK cells in the peritoneal cavity, whereas NK cell activity is ineffective against large tumors at distant sites. Although activated NK cells might not kill large tumors, NK cells activated by GM-CSF might secrete Th1 type cytokines such as interferon-γ, followed by activation of T cells and macrophages, and upregulation of MHC expression. Moreover, DCs might be fully matured by simultaneous CD40L signals, resulting in the induction of cytotoxic CD8a^+^ and helper CD4^+^ T cells and their infiltration into tumor tissues. In our DNA vaccine system, CTLs are a major effector that inhibits tumor progression, because the survival benefit was completely abrogated by *in vivo* CD8a^+^ cell depletion. However, there is the possibility that anti-CD8a antibodies might deplete subsets of CD11c^+^ cells, because mouse splenic CD8a^+^ CD11c^−^ lineage phenotype (Lin) cells can differentiate into CD8a^+^ DCs *in vivo* [34]. Secondly, the complete rejection of re-challenged tumor cells by the SART3/CD40L+GM-CSF vaccine, even in 40% of recipient mice, indicates the possibility of development of SART3-specific memory CD4^+^ T cells. Such memory cells were verified by the fact that CD4^+^ cell depletion *in vivo* almost completely inhibited the anti-tumor effect, which was similar to CD8a^+^ or dual CD4^+^/CD8a^+^ cell depletion.

The advantage of our DNA vaccine for clinical application was shown by the observation that the anti-tumor vaccine effect was highly induced irrespective of the different MHC haplotypes in BALB/c and CB57/BL6 mice. The potential of our DNA vaccine platform might be superior to the combined injection of several types of helper/killer-hybrid epitope long-peptide vaccines, which have been recently developed as a new generation of peptide vaccines [35], because all patients regardless of MHC haplotype are eligible for DNA vaccines. In this study, we designed the vaccination protocols to mimic the clinical settings of adjuvant therapy after surgical resection. In general, the tumor microenvironment shifts to immunosuppressive with an increase in the number of regulatory T cells (Tregs) and myeloid-derived suppressor cells (MDSCs) [36], [37] in accordance with cancer progression. However, the immunosuppressive state may vary among cancer types because a recent clinical trial reported no increase in the number of suppressor cells in pancreatic cancer [38]. It may be beneficial to co-administer anti-CTLA4/PD1 antibodies [39], [40] or a low dose of anti-cancer drugs such as cyclophosphamide or gemcitabine [41]–[43] to inhibit Treg- and MDSC-mediated immunosuppression, although there may be side effects of autoimmune-like disorders and bone marrow suppression. Taken together, subcutaneous/intradermal administration of polyplex micelles may increase the compliance and safety reserve for poorly conditioned patients with advanced malignancies, whereas the safety of i.p. administration has been partly validated in cynomolgus monkeys [15]. Further investigation of TAA transgenes, administration routes, and combinatorial therapies should be addressed for future clinical application to treat established solid tumors.

Polyplex micelle-based transfection of the SART3 gene plus adjuvant GM-CSF and CD40L genes, but not SART3 gene transfection alone, coordinately activated DCs, NK cells, and T cells in lymphatic organs. Consequently, there was increased helper T cell and CTL infiltration into tumor regions, resulting in prolongation of survival for mice with peritoneal dissemination of cancer cells and inhibition of distant lung metastasis in mice with subcutaneous tumors. We conclude that the TAA/CD40L+GM-CSF gene-loaded polyplex micelle is a simple and versatile vaccine platform to elicit anti-tumor immunity irrespective of the MHC haplotypes of recipients.

## Supporting Information

Figure S1 Western blotting of SART3 in LLC and CT26 cancer cells and normal mouse organ tissues. Protein samples were extracted from the indicated cancer cells and normal organ tissues of BALB/c mice and subjected to western blot analysis of SART3. The expression level of SART3 was remarkably increased in LLC and CT26 cancer cells but not in normal tissues.
(TIF)

Figure S2 Complex formation after mixing block/homo polymers with expression plasmids for SART3, CD40L, and GM-CSF genes. The ∼100 nm-sized particles were validated by measurement of the DLS (mean diameter  =  91.3±3.2 nm; PDI  = 0.16±0.02; n = 3).
(TIF)

Figure S3 Validation of transgene expression by administration of pDNA-loaded polyplex micelles. SUIT2 human pancreatic cancer cells were treated with mouse SART3/CD40L/GM-CSF gene-loaded polyplex micelles for 48 hours. RNA samples were extracted and mouse SART3, CD40L, and GM-CSF gene expression was confirmed by real-time RT-PCR. *P<0.01 vs. mock control (n = 4).
(TIF)

Figure S4 Flow cytometric analysis of maturation markers of CD11c-positive cells in the spleen. Splenocytes were isolated at 48 hours after the second i.p. administration of SART3/CD40L/GM-CSF gene-loaded polyplex micelles to BALB/c mice. Flow cytometry showed that the expression of maturation markers (CD80, CD86, and MHC class II) of CD11c-positive cells was significantly increased in the DNA vaccine group compared with that in the mock control. * P<0.05, ** P<0.01 vs. the mock control (n = 3).
(TIF)

## References

[1] Smits EL, Anguille S, Cools N, Berneman ZN, Van Tendeloo VF (2009) Dendritic cell-based cancer gene therapy. Hum Gene Ther 20: 1106–1118. 19656053 10.1089/hum.2009.145

[2] Andersen BM, Ohlfest JR (2012) Increasing the efficacy of tumor cell vaccines by enhancing cross priming. Cancer Lett 325: 155–164. 22809568 10.1016/j.canlet.2012.07.012 PMC3461191

[3] Lu L, Woo J, Rao AS, Li Y, Watkins SC, et al (1994) Propagation of dendritic cell progenitors from normal mouse liver using granulocyte/macrophage colony-stimulating factor and their maturational development in the presence of type-1 collagen. J Exp Med 179: 1823–1834. 8195710 10.1084/jem.179.6.1823 PMC2191530

[4] Vonderheide RH, Glennie MJ (2013) Agonistic CD40 antibodies and cancer therapy. Clin Cancer Res 19: 1035–1043. 23460534 10.1158/1078-0432.CCR-12-2064 PMC3590838

[5] van de Laar L, Coffer PJ, Woltman AM (2012) Regulation of dendritic cell development by GM-CSF: molecular control and implications for immune homeostasis and therapy. Blood 119: 3383–3393. 22323450 10.1182/blood-2011-11-370130

[6] Lazoura E, Apostolopoulos V (2005) Insights into peptide-based vaccine design for cancer immunotherapy. Curr Med Chem 12: 1481–1494. 15974981 10.2174/0929867054039017

[7] Berzofsky JA, Terabe M, Wood LV (2012) Strategies to use immune modulators in therapeutic vaccines against cancer. Semin Oncol 39: 348–357. 22595057 10.1053/j.seminoncol.2012.02.002 PMC3356566

[8] Mackiewicz J, Mackiewicz A (2009) Design of clinical trials for therapeutic cancer vaccines development. Eur J Pharmacol 625: 84–89. 19835869 10.1016/j.ejphar.2009.09.069

[9] Barth RJ Jr, Fisher DA, Wallace PK, Channon JY, Noelle RJ, et al (2010) A randomized trial of ex vivo CD40L activation of a dendritic cell vaccine in colorectal cancer patients: tumor-specific immune responses are associated with improved survival. Clin Cancer Res 16: 5548–5556. 20884622 10.1158/1078-0432.CCR-10-2138 PMC2994719

[10] Dessureault S, Alsarraj M, McCarthy S, Hunter T, Noyes D, et al (2005) A GM-CSF/CD40L producing cell augments anti-tumor T cell responses. J Surg Res 125: 173–181. 15854671 10.1016/j.jss.2004.11.036

[11] Katayose S, Kataoka K (1997) Water-soluble polyion complex associates of DNA and poly(ethylene glycol)-poly(L-lysine) block copolymer. Bioconjug Chem 8: 702–707. 9327134 10.1021/bc9701306

[12] Kakizawa Y, Kataoka K (2002) Block copolymer micelles for delivery of gene and related compounds. Adv Drug Deliv Rev 54: 203–222. 11897146 10.1016/s0169-409x(02)00017-0

[13] Miyata K, Nishiyama N, Kataoka K (2012) Rational design of smart supramolecular assemblies for gene delivery: chemical challenges in the creation of artificial viruses. Chem Soc Rev 41: 2562–2574. 22105545 10.1039/c1cs15258k

[14] Kumagai M, Shimoda S, Wakabayashi R, Kunisawa Y, Ishii T, et al (2012) Effective transgene expression without toxicity by intraperitoneal administration of PEG-detachable polyplex micelles in mice with peritoneal dissemination. J Control Release 160: 542–551. 22484197 10.1016/j.jconrel.2012.03.021

[15] Ohgidani M, Furugaki K, Shinkai K, Kunisawa Y, Itaka K, et al (2013) Block/homo polyplex micelle-based GM-CSF gene therapy via intraperitoneal administration elicits antitumor immunity against peritoneal dissemination and exhibits safety potentials in mice and cynomolgus monkeys. J Control Release 167: 238–247. 23422727 10.1016/j.jconrel.2013.02.006

[16] Chen Q, Osada K, Ishii T, Oba M, Uchida S, et al (2012) Homo-catiomer integration into PEGylated polyplex micelle from block-catiomer for systemic anti-angiogenic gene therapy for fibrotic pancreatic tumors. Biomaterials 33: 4722–4730. 22444644 10.1016/j.biomaterials.2012.03.017

[17] Miyata K, Oba M, Nakanishi M, Fukushima S, Yamasaki Y, et al (2008) Polyplexes from poly(aspartamide) bearing 1,2-diaminoethane side chains induce pH-selective, endosomal membrane destabilization with amplified transfection and negligible cytotoxicity. J Am Chem Soc 130: 16287–16294. 19006313 10.1021/ja804561g

[18] Itaka K, Ishii T, Hasegawa Y, Kataoka K (2010) Biodegradable polyamino acid-based polycations as safe and effective gene carrier minimizing cumulative toxicity. Biomaterials 31: 3707–3714. 20153891 10.1016/j.biomaterials.2009.11.072

[19] Uchida S, Itaka K, Chen Q, Osada K, Ishii T, et al (2012) PEGylated polyplex with optimized PEG shielding enhances gene introduction in lungs by minimizing inflammatory responses. Mol Ther 20: 1196–1203. 22334020 10.1038/mt.2012.20 PMC3369293

[20] Yang D, Nakao M, Shichijo S, Sasatomi T, Takasu H, et al (1999) Identification of a gene coding for a protein possessing shared tumor epitopes capable of inducing HLA-A24-restricted cytotoxic T lymphocytes in cancer patients. Cancer Res 59: 4056–4063. 10463607

[21] Minami T, Matsueda S, Takedatsu H, Tanaka M, Noguchi M, et al (2007) Identification of SART3-derived peptides having the potential to induce cancer-reactive cytotoxic T lymphocytes from prostate cancer patients with HLA-A3 supertype alleles. Cancer Immunol Immunother 56: 689–698. 16937115 10.1007/s00262-006-0216-9 PMC11030603

[22] Ito M, Shichijo S, Miyagi Y, Kobayashi T, Tsuda N, et al (2000) Identification of SART3-derived peptides capable of inducing HLA-A2-restricted and tumor-specific CTLs in cancer patients with different HLA-A2 subtypes. Int J Cancer 88: 633–639. 11058882 10.1002/1097-0215(20001115)88:4<633::aid-ijc18>3.0.co;2-n

[23] Harada K, Yamada A, Mine T, Kawagoe N, Takasu H, et al (2000) Mouse homologue of the human SART3 gene encoding tumor-rejection antigen. Jpn J Cancer Res 91: 239–247. 10761712 10.1111/j.1349-7006.2000.tb00937.x PMC5926322

[24] Furugaki K, Pokorna K, Le Pogam C, Aoki M, Reboul M, et al (2010) DNA vaccination with all-trans retinoic acid treatment induces long-term survival and elicits specific immune responses requiring CD4+ and CD8+ T-cell activation in an acute promyelocytic leukemia mouse model. Blood 115: 653–656. 19965687 10.1182/blood-2007-08-109009

[25] Jedema I, van der Werff NM, Barge RM, Willemze R, Falkenburg JH (2004) New CFSE-based assay to determine susceptibility to lysis by cytotoxic T cells of leukemic precursor cells within a heterogeneous target cell population. Blood 103: 2677–2682. 14630824 10.1182/blood-2003-06-2070

[26] Hirano K, Hunt CA (1985) Lymphatic transport of liposome-encapsulated agents: effects of liposome size following intraperitoneal administration. J Pharm Sci 74: 915–921. 4067845 10.1002/jps.2600740902

[27] Un K, Kawakami S, Suzuki R, Maruyama K, Yamashita F, et al (2010) Enhanced transfection efficiency into macrophages and dendritic cells by a combination method using mannosylated lipoplexes and bubble liposomes with ultrasound exposure. Hum Gene Ther 21: 65–74. 19719400 10.1089/hum.2009.106

[28] Slingluff CL, Petroni GR, Smolkin ME, Chianese-Bullock KA, Smith K, et al (2010) Immunogenicity for CD8+ and CD4+ T cells of 2 formulations of an incomplete freund's adjuvant for multipeptide melanoma vaccines. J Immunother 33: 630–638. 20551833 10.1097/CJI.0b013e3181e311ac PMC3218563

[29] Kuwajima S, Sato T, Ishida K, Tada H, Tezuka H, et al (2006) Interleukin 15–dependent crosstalk between conventional and plasmacytoid dendritic cells is essential for CpG-induced immune activation. Nat Immunol 7: 740–746. 16715101 10.1038/ni1348

[30] Zhang M, Obata C, Hisaeda H, Ishii K, Murata S, et al (2005) A novel DNA vaccine based on ubiquitin-proteasome pathway targeting 'self'-antigens expressed in melanoma/melanocyte. Gene Ther 12: 1049–1057. 15800663 10.1038/sj.gt.3302490

[31] Ciupitu AM, Petersson M, O'Donnell CL, Williams K, Jindal S, et al (1998) Immunization with a lymphocytic choriomeningitis virus peptide mixed with heat shock protein 70 results in protective antiviral immunity and specific cytotoxic T lymphocytes. J Exp Med 187: 685–691. 9480978 10.1084/jem.187.5.685 PMC2212166

[32] Zarei S, Schwenter F, Luy P, Aurrand-Lions M, Morel P, et al (2009) Role of GM-CSF signaling in cell-based tumor immunization. Blood 113: 6658–6668. 19282460 10.1182/blood-2008-06-161075 PMC2943756

[33] Ma DY, Clark EA (2009) The role of CD40 and CD154/CD40L in dendritic cells. Semin Immunol 21: 265–272. 19524453 10.1016/j.smim.2009.05.010 PMC2749083

[34] Wang Y, Zhang Y, Yoneyama H, Onai N, Sato T, et al (2002) Identification of CD8alpha+CD11c- lineage phenotype-negative cells in the spleen as committed precursor of CD8alpha+ dendritic cells. Blood 100: 569–577. 12091350 10.1182/blood.v100.2.569

[35] Takahashi N, Ohkuri T, Homma S, Ohtake J, Wakita D, et al (2012) First clinical trial of cancer vaccine therapy with artificially synthesized helper/killer-hybrid epitope long peptide of MAGE-A4 cancer antigen. Cancer Sci 103: 150–153. 22221328 10.1111/j.1349-7006.2011.02106.x PMC11164142

[36] Clark CE, Hingorani SR, Mick R, Combs C, Tuveson DA, et al (2007) Dynamics of the immune reaction to pancreatic cancer from inception to invasion. Cancer Res 67: 9518–9527. 17909062 10.1158/0008-5472.CAN-07-0175

[37] Pan PY, Wang GX, Yin B, Ozao J, Ku T, et al (2008) Reversion of immune tolerance in advanced malignancy: modulation of myeloid-derived suppressor cell development by blockade of stem-cell factor function. Blood 111: 219–228. 17885078 10.1182/blood-2007-04-086835 PMC2200807

[38] Lutz E, Yeo CJ, Lillemoe KD, Biedrzycki B, Kobrin B, et al (2011) A lethally irradiated allogeneic granulocyte-macrophage colony stimulating factor-secreting tumor vaccine for pancreatic adenocarcinoma. A Phase II trial of safety, efficacy, and immune activation. Ann Surg 253: 328–335. 21217520 10.1097/SLA.0b013e3181fd271c PMC3085934

[39] Wing K, Onishi Y, Prieto-Martin P, Yamaguchi T, Miyara M, et al (2008) CTLA-4 control over Foxp3+ regulatory T cell function. Science 322: 271–275. 18845758 10.1126/science.1160062

[40] Wang W, Lau R, Yu D, Zhu W, Korman A, et al (2009) PD1 blockade reverses the suppression of melanoma antigen-specific CTL by CD4+ CD25(Hi) regulatory T cells. Int Immunol 21: 1065–1077. 19651643 10.1093/intimm/dxp072 PMC2731790

[41] Tongu M, Harashima N, Monma H, Inao T, Yamada T, et al (2013) Metronomic chemotherapy with low-dose cyclophosphamide plus gemcitabine can induce anti-tumor T cell immunity in vivo. Cancer Immunol Immunother 62: 383–391. 22926062 10.1007/s00262-012-1343-0 PMC11029128

[42] Le DT, Jaffee EM (2012) Regulatory T-cell modulation using cyclophosphamide in vaccine approaches: a current perspective. Cancer Res 72: 3439–3444. 22761338 10.1158/0008-5472.CAN-11-3912 PMC3399042

[43] Shevchenko I, Karakhanova S, Soltek S, Link J, Bayry J, et al (2013) Low-dose gemcitabine depletes regulatory T cells and improves survival in the orthotopic Panc02 model of pancreatic cancer. Int J Cancer 133: 98–107. 23233419 10.1002/ijc.27990

