# Supplementary figures and images for: Intraperitoneal Administration of a Tumor-Associated Antigen SART3, CD40L, and GM-CSF Gene-Loaded Polyplex Micelle Elicits a Vaccine Effect in Mouse Tumor Models
Source: PLoS One. 2014 Jul 11;9(7):e101854. doi: 10.1371/journal.pone.0101854 (PMC4094388; doi:10.1371/journal.pone.0101854)

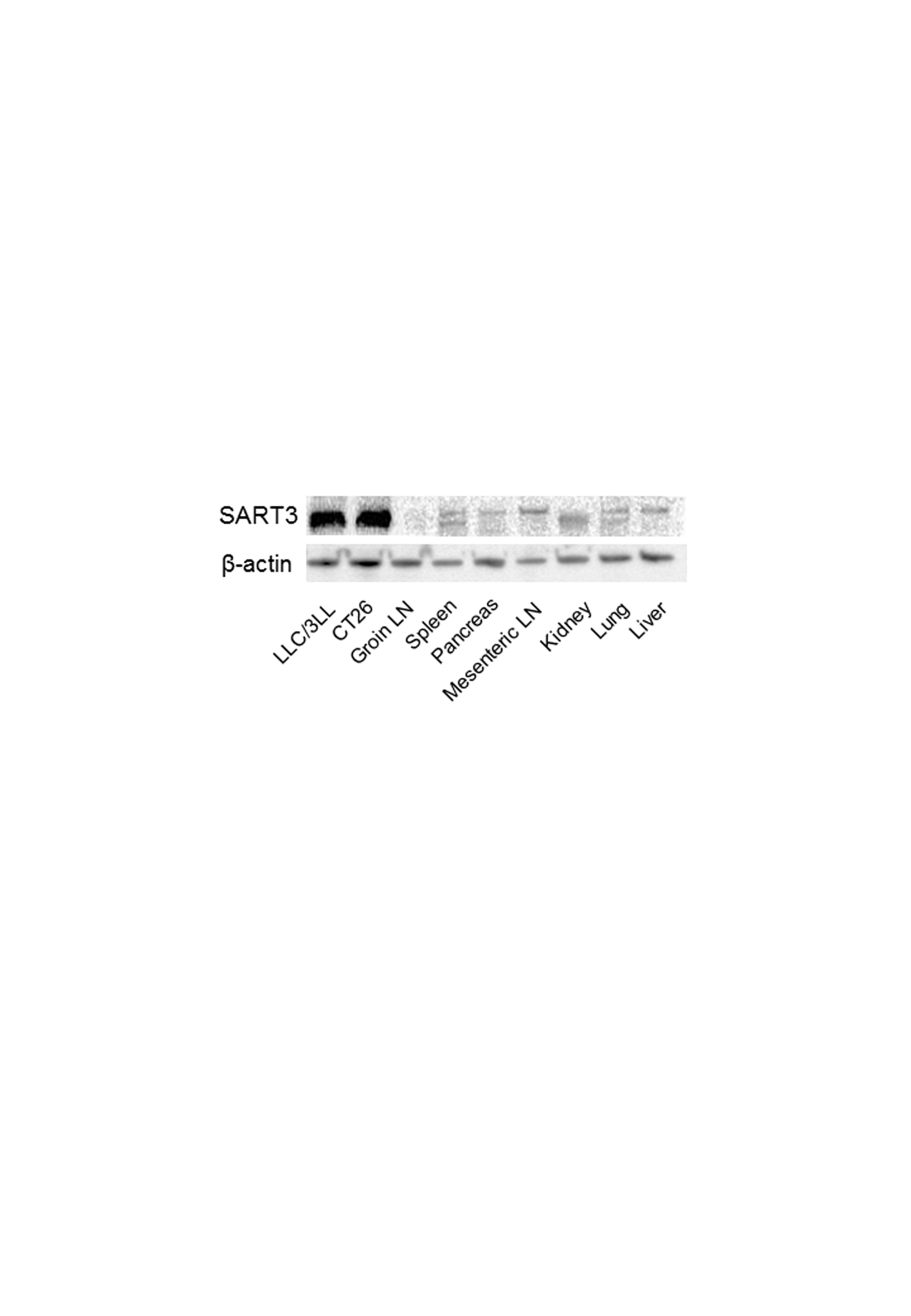

Supplement: Figure S1 — Western blotting of SART3 in LLC and CT26 cancer cells and normal mouse organ tissues. Protein samples were extracted from the indicated cancer cells and normal organ tissues of BALB/c mice and subjected to western blot analysis of SART3. The expression level of SART3 was remarkably increased in LLC and CT26 cancer cells but not in normal tissues. (TIF) [file pone.0101854.s001.tif]

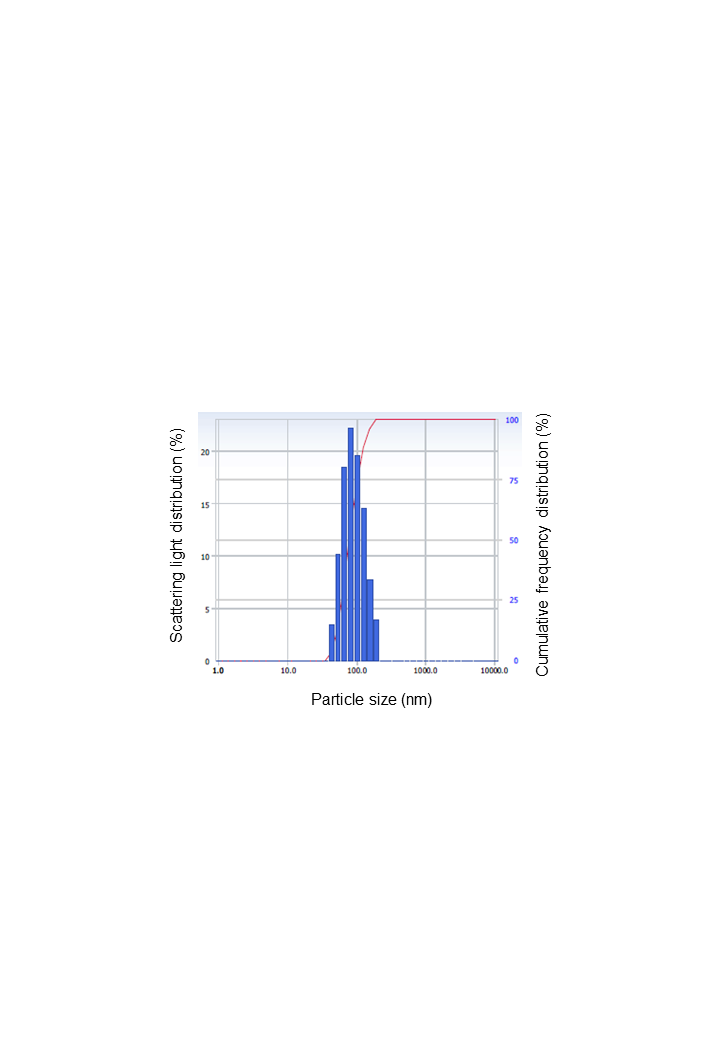

Supplement: Figure S2 — Complex formation after mixing block/homo polymers with expression plasmids for SART3, CD40L, and GM-CSF genes. The ∼100 nm-sized particles were validated by measurement of the DLS (mean diameter = 91.3±3.2 nm; PDI = 0.16±0.02; n = 3). (TIF) [file pone.0101854.s002.tif]

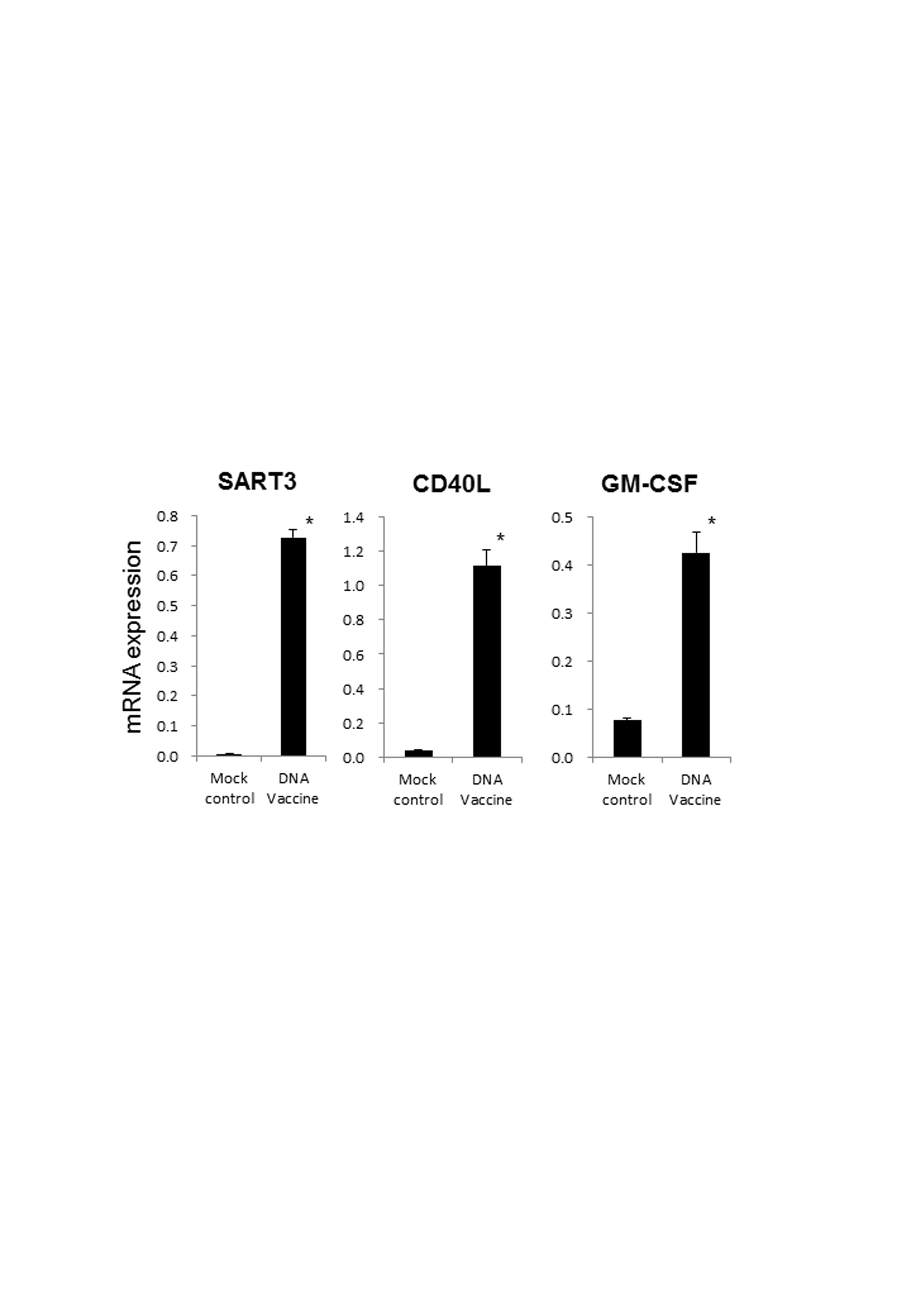

Supplement: Figure S3 — Validation of transgene expression by administration of pDNA-loaded polyplex micelles. SUIT2 human pancreatic cancer cells were treated with mouse SART3/CD40L/GM-CSF gene-loaded polyplex micelles for 48 hours. RNA samples were extracted and mouse SART3, CD40L, and GM-CSF gene expression was confirmed by real-time RT-PCR. *P<0.01 vs. mock control (n = 4). (TIF) [file pone.0101854.s003.tif]

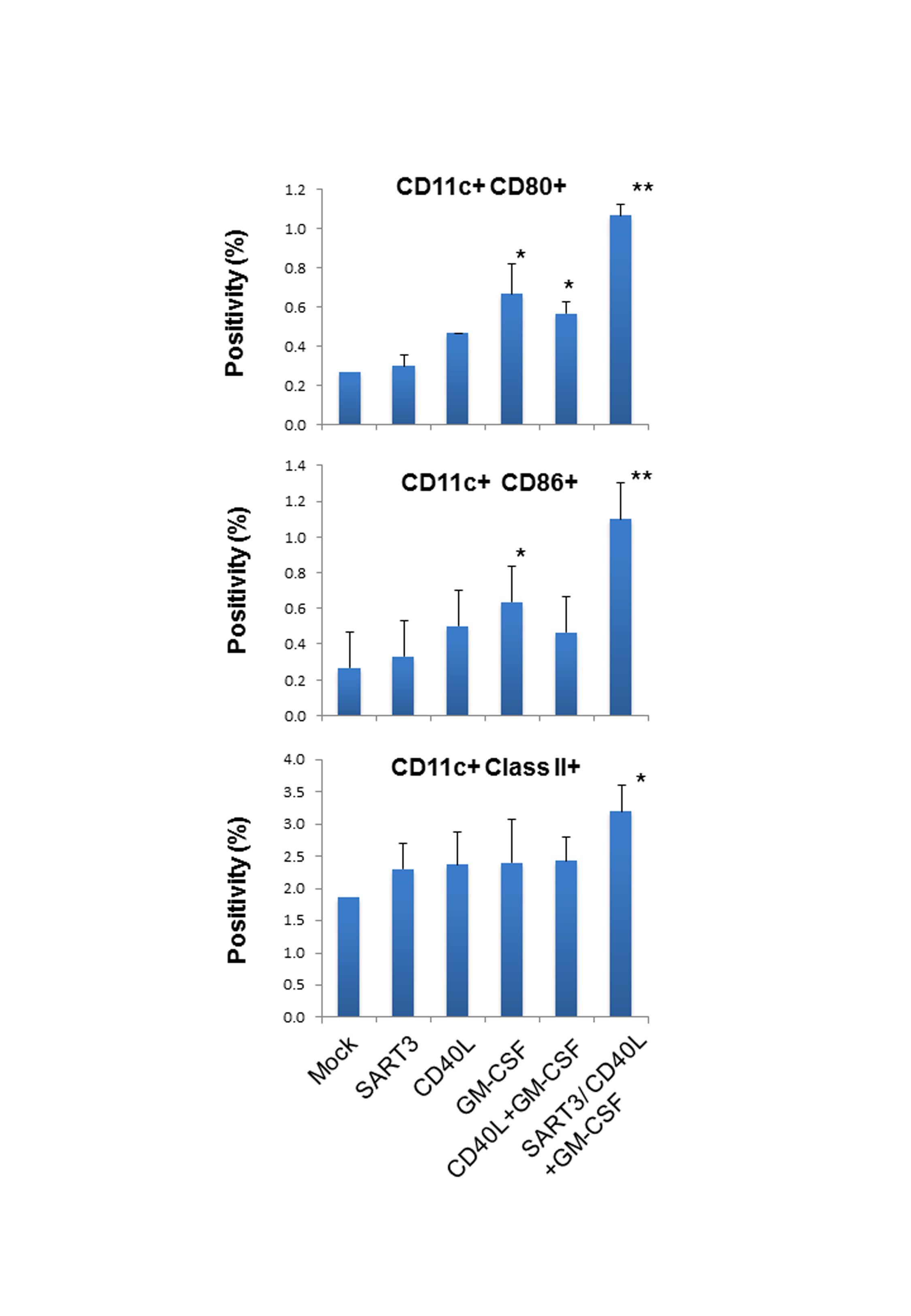

Supplement: Figure S4 — Flow cytometric analysis of maturation markers of CD11c-positive cells in the spleen. Splenocytes were isolated at 48 hours after the second i.p. administration of SART3/CD40L/GM-CSF gene-loaded polyplex micelles to BALB/c mice. Flow cytometry showed that the expression of maturation markers (CD80, CD86, and MHC class II) of CD11c-positive cells was significantly increased in the DNA vaccine group compared with that in the mock control. * P<0.05, ** P<0.01 vs. the mock control (n = 3). (TIF) [file pone.0101854.s004.tif]
